# Adiponectin as Link Factor between Adipose Tissue and Cancer

**DOI:** 10.3390/ijms20040839

**Published:** 2019-02-15

**Authors:** Erika Di Zazzo, Rita Polito, Silvia Bartollino, Ersilia Nigro, Carola Porcile, Andrea Bianco, Aurora Daniele, Bruno Moncharmont

**Affiliations:** 1 Department of Medicine and Health Sciences “V. Tiberio”, University of Molise, Campobasso 86100, Italy; erika.dizazzo@unimol.it (E.D.Z.); silvia.bartollino@unimol.it (S.B.); carola.porcile@unimol.it (C.P.); 2 Department of Precision Medicine, University of Campania “Luigi Vanvitelli”, Naples 80131, Italy; 3 Dipartimento di Scienze e Tecnologie Ambientali Biologiche Farmaceutiche, Università degli Studi della Campania “Luigi Vanvitelli”, Caserta 81100, Italy; rita.polito@unicampania.it (R.P.); nigro@ceinge.unina.it (E.N.); 4 CEINGE-Biotecnologie Avanzate Scarl, Napoli 80145, Italy; 5 Dipartimento di Scienze Cardio-Toraciche e Respiratorie, Università degli Studi della Campania “Luigi Vanvitelli”, Napoli 80131, Italy; andrea.bianco@unimol.it

**Keywords:** Adiponectin, cancer, Adiponectin receptors, obesity, inflammatory response, inflammation, nutritional status

## Abstract

Adipose tissue is a key regulator of energy balance playing an active role in lipid storage as well as in synthesizing several hormones directly involved in the pathogenesis of obesity. Obesity represents a peculiar risk factor for a growing list of cancers and is frequently associated to poor clinical outcome. The mechanism linking obesity and cancer is not completely understood, but, amongst the major players, there are both chronic low-grade inflammation and deregulation of adipokines secretion. In obesity, the adipose tissue is pervaded by an abnormal number of immune cells that create an inflammatory environment supporting tumor cell proliferation and invasion. Adiponectin (APN), the most abundant adipokine, shows anti-inflammatory, anti-proliferative and pro-apoptotic properties. Circulating levels of APN are drastically decreased in obesity, suggesting that APN may represent the link factor between obesity and cancer risk. The present review describes the recent advances on the involvement of APN and its receptors in the etiology of different types of cancer.

## 1. Introduction

Obesity, characterized by an excessive and chronic fat accumulation harmful to health, is defined by the World Health Organization (WHO) as a body mass index (BMI) of 30 or more and represents a worldwide emergent public health problem [1]. Several epidemiological studies revealed an alarming increase in the number of obese individuals in recent decades, reaching epidemic distribution in many areas of the World [2]. Currently, more than 1.5 billion adults are overweight and about 600 million people are classified as obese worldwide and these rates are estimated to increase in the future [3]. Obesity is a major risk factor for the development of metabolic and cardiovascular diseases as well as for several malignancies, frequently associated to a poor clinical outcome [4,5,6]. Cancer is the second leading cause of death worldwide; consequently, new efforts are needed to understand how obesity induce the cancer onset and affects its outcomes. The hypothesis that adipose tissue is involved in tumorigenesis is now called “adiponcosis” [7]. Molecular mechanisms linking obesity and cancer are complex and still not fully clarified. A low-grade chronic inflammation, deregulation of growth signaling pathways, chronic hyperinsulinemia and obesity-associated hypoxia are widely accepted to be pivotal factors in cancer pathogenesis [8]. Adipose tissue, originally defined as a passive fat depot, is a heterogeneous tissue, strongly committed to energy substrate homeostasis but endowed with complex secretory functions related to the nutritional status, thereby recognized as an active endocrine organ [9]. It produces and secretes different bioactive molecules, called adipokines. In obesity, the alteration of endocrine functions of adipose tissue negatively affects the secretion of different adipocytokines. Among them, Adiponectin (APN), the most adipocyte secretory protein, shows a reduced expression levels in obesity [10]. The reduction of APN expression levels observed in obese patients, has been related to an increase of tumor onset risk. Several studies demonstrated that APN, beyond its actions in metabolic responses such as energy metabolism regulation and insulin-sensitivity, has pleiotropic effects in cancer. Although literature data on the role of APN in carcinogenesis is conflicting, the most accredited hypothesis is that APN has a protective role, such as anti-inflammatory, anti-proliferative and pro-apoptotic effects, avoiding the development and progression of several malignancies, such as breast, colon, prostate, liver and endometrial cancers [11,12,13,14].

The aim of this review is to appraise the role of APN and its molecular pathways in “big killer” cancers, such as breast, colon, thyroid and lung cancer.

## 2. Adiponectin Structure and Function

First described in 1995, APN, also named adipocyte complement-related protein (Acrp30, *ADIPOQ*, apM1, GBP28), represents the most relevant insulin-sensitizing adipokine, primarily controlling glucose uptake as well as stimulating fatty acids oxidation [15]. APN is encoded by the *ADIPOQ* gene, which spans approximately 15.8 kb and is structured in three exons on chromosome 3q27; this region has been linked to a susceptibility locus for metabolic syndrome, type 2 diabetes and cardiovascular disease [16]. APN is mainly produced from adipose tissue but it is released at much lower concentration from other tissues [14]. Full-length APN is a 30-kDa protein with a primary sequence of 244 amino acids, composed of four domains: a signal sequence (aa 1–18), a non-conserved N-terminal domain (aa 19–41), followed by a 22 collagen-like repeat domain (aa 42–107) and a C-terminal globular domain (aa 108–244). By the cleavage of full-length APN, the globular APN (gAPN), containing only the C-terminal domain is obtained. APN can exist as different oligomers: trimers (approx. 90 kDa basic unit; Low Molecular Weight, LMW), hexamers (approx. 180 kDa, Medium Molecular Weight, MMW) and multimers (approx. 360–400 kDa, High Molecular Weight, HMW) [14]. 

The APN correct folding starts with trimers formation that, through the collagenous domains, assemble into hexamers (MMW); subsequently, these primordial complexes associate into multimers, (HMW), the most biologically active form [14]. APN biological activity depends strictly on its structure assembly, determined by post-translational modifications [17]. In particular, post-translational modifications of the oligomeric forms, involving hydroxylation and glycosylation of four conserved lysine residues at positions 68, 71, 80 and 104 on the collagenous domain, determine the formation of the high-molecular weight (HMW) complex APN [18]. Impairment of APN oligomers formation has an impact on insulin concentration, liver gluconeogenesis and can induce severe cardio-metabolic dysfunctions [17,18]. Furthermore, Arg112Cys and Ile164Thr mutations in the APN protein, preventing the trimer assembly, cause an impaired cellular secretion and are clinically associated with hypoadiponectinemia [19]. In physiological conditions, APN is an abundant protein in systemic circulation, representing about 0.01% of the total serum protein, with a concentration range of 5–50 μg/mL [14,20]. The APN serum concentration is inversely related to BMI and to insulin resistance [10,21]. However, in pathological conditions characterized by a chronic inflammation, such as type 2 diabetes, obesity and atherosclerosis, a lowering in APN serum concentrations is observed [10,20,22]. APN mediates most of its biological effects by binding to its classical receptors, AdipoR1 and AdipoR2, belonging to seven-transmembrane domains receptor family. Both receptors have been detected in almost normal and cancer tissues. AdipoR1 shows higher affinity for the globular protein than the full-length APN molecule, while AdipoR2 has a similar affinity for both forms. In obese individuals, a reduction in AdipoR1 and AdipoR2 expression levels seems to lead to a decreased sensitivity to APN [23]. Additionally, hexameric and multimeric APN bind the third non- classical receptor recognized, the glycosylphosphatidyl inositol (GPI)-anchored T-cadherin receptor [24].

## 3. Adiponectin Signaling Pathways

Several lines of evidence suggest that APN upon binding to its receptors, induces the recruitment of the adaptor protein APPL1, thereby activating a plethora of downstream signaling pathways controlling cell survival, cell growth and apoptosis. APN effects are mostly mediated via AMPK, mTOR, PI3K/AKT, MAPK, STAT3 and NF-kB [12]. APN induces the activation of AMPK, a central sensor and regulator of cellular energy, that in turn stimulates the expression of p21 and p53 and phosphorylates p53 to initiate cell cycle arrest, senescence and apoptosis. Additionally, studies point toward the inhibitory effects of APN on the PI3K/AKT/mTOR pathways, which leads to a cascade of events resulting in a blockade of cell survival, growth and proliferation. APN signaling also activates the MAPK cascade, which involves cJNK, p38 and ERK1/2. The cJNK and p38 action on proliferation and apoptosis depend on the cell type, whereas ERK1/2 have frequently a mitogenic effect. APN inhibits STAT3 activation that increases tumor cell proliferation, survival, angiogenesis and invasion, as well as inhibiting anti-tumor immunity. APN, through the suppression of inhibitor of NF-kB phosphorylation, suppresses the pro-inflammatory and anti-apoptotic NF-kB pathway [14].

## 4. Adipose Tissue, Adiponectin and Low Chronic Inflammation

In adipose tissue there is a perfect balance between adipocytes and immune cells that is lost in obesity, leading to a local chronic low inflammation associated with increased cancer risk. Immune cells infiltrating the adipose tissue of obese patients regulate the local immune responses, by increasing the levels of pro-inflammatory cytokines and adipokines thus supporting tumor development. Clusters of enlarged adipocytes become distant from the blood vessels, leading to a local area of hypoxia that underlies the inflammatory response [25,26]. Several immune cell types are involved in the development of adipose tissue inflammation: neutrophils and mast cells have been implicated in promoting inflammation and insulin resistance in obesity, whereas eosinophils and myeloid-derived suppressor cells have been suggested to play a protective role. In addition, a prominent role of B- and T-lymphocytes and natural-killer cells in adipose tissue inflammation recently emerged [27]. The cross-talk between adipocytes and cancer cells is mediated by cytokines (specifically IL-1, IL-6 and TNF-α), adipokines, including APN and other molecules, released by adipose tissue, able to control proliferation and invasion of different cancer cell types [28]. APN suppresses immune cell proliferation and, in particular, proliferation and polarization of type 1 macrophages (pro-inflammatory phenotype) while inducing proliferation and polarization of type 2 macrophages (anti-inflammatory phenotype). APN additionally reduces B-cells lymphopoiesis and T-cells responsiveness. Finally, APN acting on inflammatory response suppresses the expression of several pro-inflammatory mediators, such as TNF-α [29].

## 5. Adiponectin in Cancer

**Figure 1:**
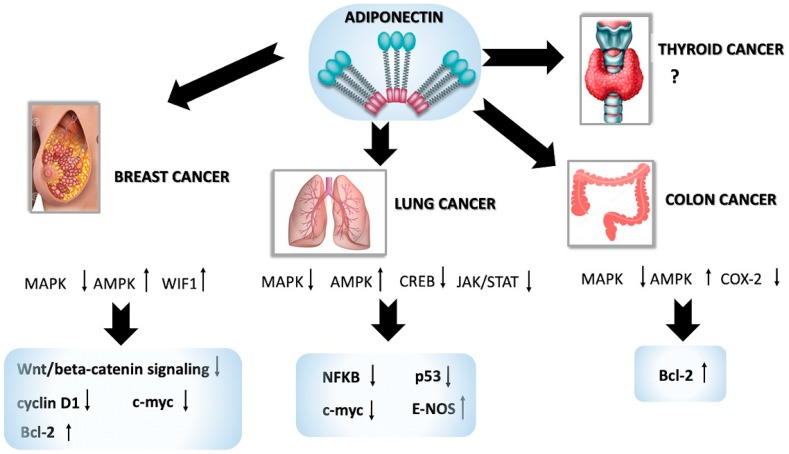
Summary of the molecular mechanisms affected by APN in cancers.

Although literature data on the role of APN in carcinogenesis are conflicting, it is recognized that APN is able to reduce development and progression of several malignancies, such as breast, colon, lung, thyroid and other cancers, through different molecular mechanisms, which are described below (see Figure 1).

### 5.1. Adiponectin in Breast Cancer

Breast cancer (BC) is a well-known obesity-related cancer [30]. Adipose tissue may increase BC risk via a dual mechanism: (i) aromatization of adrenal androgens to estrogens in the adipocyte increases estrogen circulating levels and consequently promoting proliferation of mammary epithelial cells; (ii) deregulation of adipokine’s expression and secretion, thus reducing the APN anti-proliferative effect on breast cells [31]. Recent evidence demonstrates that low APN serum levels are associated with increased BC risk but the precise APN mechanism of action is not completely understood [32]. Depending on tumor phenotype (ERα-positive or negative) and through the cooperation with circulating or locally-produced growth factors, APN affects BC cell growth, aggressiveness and behavior [33]. In the ERα-negative human BC cell line MDA-MB-231, APN elicits an anti-proliferative effect by modulating the expression of genes controlling cell cycle progression, such as p53, Bax, Bcl-2, c-myc and cyclin D1. In these cells APN inhibits PI3K/AKT pathway and activates AMPK, that in turn phosphorylates Sp1 protein. Phosphorylated Sp1 binds cyclin D1 promoter, causing the displacement of RNA Polymerase II and the recruitment of a co-repressor complex containing SMRT, NCoR and HDAC1, with a consequent repression of cyclin D1 expression and BC growth blockage [33]. In MDA-MB-231 cells and in nude mice APN also negatively controls Wnt/beta-catenin signaling, by positively regulating the expression of the Wnt inhibitory factor-1 (WIF1), a Wnt antagonist, at gene and protein levels [34].

On the other hand, conflicting results have been reported on the effects induced by APN in ERα-positive BC cells. In MCF-7 BC cell line (ERα-positive), APN is able to activate ERα that in turn stimulates cell growth [33]. Moreover, ADP400, a synthetic peptide modulating cellular APN receptor responses, induces mitogenic effects in MCF-7 cells, probably antagonizing endogenous APN actions or acting as an inverse agonist [35]. Emerging evidence shows the existence of a cross-talk between APN/AdipoR1, IGF-IR and ERα in BC [36]. Notoriously, insulin stimulates proliferation of BC cells through the IGF-1 receptors by activating PI3K [37,38,39]. In BC an increase in circulating insulin and estrogen concentration are observed together with a reduction in APN expression level [33]. Furthermore, low APN levels increase the risk of postmenopausal BC and of ER-positive breast tumors through a combined mitogenic effect of hyperinsulinemia and increased IGFs and estrogen levels. It has been demonstrated that incubation of MCF-7 cells with low APN concentrations enhances the association of IGF-1R with AdipoR1, APPL1, ERα, IGF-IR and c-Src; leading, via c-Src, to MAPK activation. The activated MAPK phosphorylates both Sp1 and ERα, allowing their recruitment on cyclin D1 promoter together with an enhanced association of RNA Polymerase II and pCAF. This leads to an increased cyclin D1 expression, inducing BC growth. Such effect is abrogated in the presence of specific RNA silencers targeting ERα or IGF-1R [36,40]. On the other hand, in ERα-negative MDA-MB-231 cells, APN is unable to induce MAPK phosphorylation. Additional data demonstrates that APN is able to regulate BC cell migration and invasion [41,42]. Indeed, a positive correlation between lympho-vascular and vascular invasion and AdipoR2 but not AdipoR1, expression has been reported [43]. Additionally, the expression of both APN and AdipoRs was significantly higher in invasive BC than noninvasive cases [42].

### 5.2. Adiponectin in Lung Cancer 

Lung cancer is a highly-prevalent malignant carcinoma, representing one of the principal causes of morbidity and death worldwide [44].

To date, the molecular association between obesity and lung cancer remains still unclear and somewhat contradictory [45,46]. In fact, a meta-analysis suggested that overweight and obesity are protective factors against lung cancer, whereas other evidence indicates that obesity, in particular at visceral level, represents an important risk factor for lung cancer [45]. A variation of APN expression level was measured in patients affected by lung cancer, even though with contrasting results [47]. Some studies reported that there is no significant association between APN levels and lung cancer [48,49]. Other studies described a lowering in APN concentrations during lung cancer progression [50]. A further study revealed that APN deficiency significantly inhibited tumor vascularization and increased apoptosis and hypoxia, while APN-null mice showed a higher number of pulmonary metastases [51]. Additionally, two APN-gene promoter polymorphisms, Rs266730 and Rs2241766, have been associated with lung cancer risk and poor prognosis after surgery [52]. 

The APN inhibitory effects on lung cancer cell proliferation and invasion accompanied by an apoptosis rate increase has been mainly linked to the activation of pAMPK/mTOR pathways [53]. Moreover, APN, may exert an anti-proliferative effects through CREB down-regulation [54]. Indeed, in this recent paper, the authors reported that physiological concentrations of APN significantly reduced cell proliferation of human lung adenocarcinoma cell line A549, mainly by altering cell cycle kinetics and through the inhibition of CREB [54]. 

Beyond the inhibition of cell proliferation, a role of APN in regulating inflammation, cell growth and oxidative stress could also be observed in lung cancer cell lines. For instance, APN was effective in reducing the activation of inflammatory pathways, especially through the NF-κB-AdipoR1 pathway. Additionally, APN increased the levels of the anti-inflammatory IL-10 without influencing the expression of pro-inflammatory IL-6, IL-8 and MCP-1 in both IL-1β and TNF-α-treated A549 cells [53]. However, a pro-inflammatory role for APN has also been proposed; in fact, a report showed that APN promoted lung inflammation, via up-regulating cPLA2 and COX-2 expression together with intracellular ROS production [55]. 

Taken together, the current evidence indicates that APN and its receptors may act as molecular mediators in lung cancer at multiple levels although their role is controversial and far from being fully defined.

### 5.3. Adiponectin in Colon Cancer

Colorectal cancer (CRC) is one of the most common obesity-related cancer [56]. To date, several epidemiological and in vivo studies have investigated the role of APN in CRC [57]. Low APN serum concentration has been strongly associated to an increased risk of colorectal adenoma or early CRC [58,59,60,61]. In addition, patients affected by colon carcinoma or advanced adenoma showed markedly low APN serum level but no difference in serum APN concentration was observed between patients with advanced adenoma and patients with CRC [62].

AdipoR1 and AdipoR2 are expressed in CRC and are associated with lymph node involvement. Furthermore, AdipoR1 expression is correlated to tumor size during the early stages of CRC [61,63]. On the contrary, T-cadherin gene is not expressed in this cancer type since its promoter is frequently methylated [64,65]. 

Choe et al. using The Cancer Genome Atlas for CRC, investigated the association between the adipokine gene family (*ADIPOQ, ADIPOR1, ADIPOR2, LEP, LEPR, RETN, RETNLB, RBP4, SFRP5, NAMPT, SPP1*) mRNA expression levels and the survival rate of CRC patients, observing that a high expression level of the *ADIPOR1* and *SPP1* genes had unfavorable outcomes on CRC patients. Also, the *SPP1* mRNA expression level was significantly associated to the T- and N-stage, overall stage and mortality [66].

Evidence demonstrated that APN may affect CRC cell proliferation, adhesion, invasion as well as inflammation. Most of the studies demonstrated that APN could reduce cell proliferation rate [67,68]. Specifically, APN directly inhibited CRC cell proliferation via AdipoR1- and AdipoR2-mediated AMPK activation [30,67]. Activated AMPK, in turn, regulated many molecular mechanisms responsible for the APN inhibition of cell proliferation. Indeed, AMPK upregulated T-cadherin mRNA expression in a dose-dependent manner in HCT116 cells [69,70,71].

In vitro assay and immunohistochemical staining suggested that APN could prevent CRC carcinogenesis and proliferation by downregulating COX-2 expression [61,72]. APN treatment reduced the survival rate of both CaCo-2 and HCT116 cell lines in a time- and dose-dependent manner by inducing the phosphorylation of ERK1/2 and the cleavage of Caspase-3 thus activating programmed cell death [73]. 

In a mouse model, Saxena et al. proposed the use of APN as a therapeutic compound to decrease the severity of the symptoms caused by chronic inflammation-induced by CRC [74]. Additionally, a very recent study in a CRC patient cohort strongly suggested that the mRNA expression levels of APN and its receptors could be used as biomarkers for the prediction of CRC survival prediction [66]. On the other hand, Ogunwobi et al. reported a pro-proliferative and pro-inflammatory APN action on CRC cells [75]. This discrepancy might be explained looking at recent-published data showing that the effect of APN on cancer cell proliferation is glucose-dependent, whereby APN supports CRC survival in a low glucose medium but inhibits proliferation under a high glucose conditions [76].

Taken together these results suggest that, although APN could be an attractive target for obesity-associated colon cancer, further investigations are needed to completely elucidate the potential actions of APN in these cancers.

### 5.4. Adiponectin in Thyroid Cancer

The incidence of thyroid cancer (TC) underwent such a remarkable worldwide increase that it becomes the second most commonly diagnosed cancer in young women; nevertheless, the mechanisms underlying the development and progression of TC are poorly understood. Epidemiologic studies reported that an increase in BMI and obesity with low levels of circulating APN, are positively associated to TC risk [77]. On the contrary, Abooshahab et al. found no differences in APN levels between TC patients and healthy controls [78]. TC tissues and cells lines express both AdipoR1 and AdipoR2 [79]. A weak expression of AdipoR1 and a moderate expression of AdipoR2 were observed in both K1 and B-CPAP TC cell lines where APN stimulates AMPK phosphorylation. BHP7 and SW579 cell lines express both AdipoR1 and AdipoR2 but are not responsive to APN [79]. APN increases the synthesis of thyroid hormones, especially free thyroxine (fT4), through the interaction between the C-terminal globular domain of APN and the gC1q receptor [80].

Several molecular pathways link obesity to TC. An in vivo study performed in a TC mouse model reported that a high-fat diet (HFD) could increase cell proliferation via two main molecular pathways: i. increasing the protein levels of cyclin D1 and retinoblastoma protein (pRb) phosphorylation; ii. through chronic activation of the JAK2/STAT3 signaling pathway and induction of STAT3 gene expression [81]. In HFD mice, the JAK2-STAT3 signaling pathway was also associated with a higher occurrence of anaplastic *foci*. Interestingly, an activation of the STAT3 pathway by APN has been discovered in fibroblasts, hepatocytes and adipocytes but, to our knowledge, there is no proof of STAT3 involvement by APN in thyroid cells [82,83]. 

Altogether this evidence suggests that APN might have an important role in the development and progression of TC, even if additional studies are necessary to fully clarify the usefulness of APN as a plausible marker or therapeutic target for TC.

### 5.5. Adiponectin in Other Cancers

Recently, in a systematic review and meta-analysis, weight loss and a physically active lifestyle were associated to a lower risk of meningiomas; in the same study, the authors also observed a correlation between obesity and glioma risk [84]. Interestingly, our research group showed that APN negatively modulated cell proliferation of human glioblastomas U87-MG and U251 cell lines, by inducing growth arrest with a G1-phase delay and a slow but persistent activation of a specific subset of ERK1/2 proteins. Moreover, we observed that APN negatively regulated Insulin-like Growth Factor 1 IGF-1 action abolishing the IGF-1-induced proliferation of U251 cells [85]. Prostate cancer is one of the most commonly diagnosed cancers in men [86]. The role of APN in this type of cancer is contradictory. In a large retrospective study, low APN expression levels were related to the onset of prostate cancer. Furthermore, the APN expression level was associated to the stage and the grade of the disease. However, other studies have found no association between APN expression level and prostate cancer [12,14]. 

## 6. Conclusions

**Figure 2:**
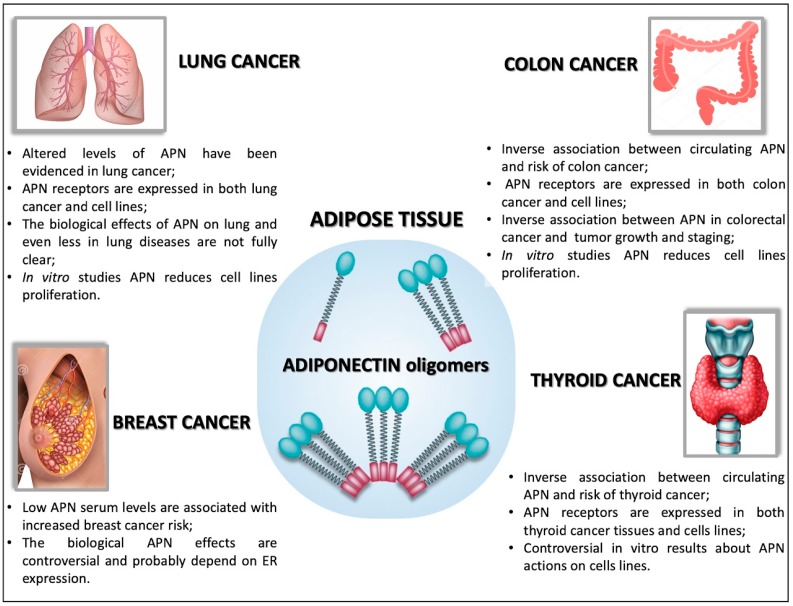
Summary of the biological functions exerted by APN in lung, colon, breast and thyroid cancers.

Obesity is a highly prevalent public health problem that has been associated with increased cancers risk in multiple organs. Several mechanisms have been proposed to explain the link between obesity and cancer and, among them, deregulation of adipocyte-secreted factors is critical. An involvement of APN, the most represented circulating adipokine, in the etiology of different cancer types has been proposed (see Figure 2). APN shows multifaceted functions in tumorigenesis. Nevertheless, the anti-proliferative and tumor-suppressor role of APN remains elusive and data collected so far are controversial. In vitro studies suggested that in a number of cancers APN may promote neoplastic growth, while in others it may suppress it. Consequently, APN likely could act both as a tumor-suppressor or as a tumor-promoting factor. The discrepancy is coherent with the evidence that APN exerts different functions depending on environmental factors, such as tissue/organ type and inflammatory state. Moreover, the available cultured-cell models probably are not suitable to reproduce the complex tumor microenvironment existing in vivo. Another level of complexity that could influence experimental results derives from the existence of different APN oligomers. Indeed, the major number of studies do not indicate which APN isoform is involved; probably this is due to the difficulty of discerning them. Moreover, alterations not only in circulating levels of total and isoform-specific APN but also in APN tumor microenvironment concentrations, remain to be explored in human specimens. Additional clues may facilitate the development of new strategies for the successful treatment of many obesity- related malignancies, such as by increasing APN serum concentration or antagonizing its receptors, and/or by targeting APN signaling pathways.

## Abbreviations

AdipoR	Adiponectin receptor
AMPK	5’-adenosine monophosphate-activated protein kinase
APN	Adiponectin
BC	Breast cancer
BMI	Body Mass Index
COX-2	Cyclooxygenase
CRC	Colorectal cancer
cPLA2	Cytosolic phospholipase A2
CREB	cAMP response element-binding protein
ER	Estrogen receptor
ERK1/2	Extracellular signal-regulated protein kinases 1 and 2
fT4	Free thyroxine
HFD	High-fat diet
IGF-1	Insulin-like growth factor 1
IL-10	Interleukin-10
IL-6	Interleukin-6
IL-8	Interleukin-8
JAK2	Janus kinase 2
MAPK	Mitogen-activated protein kinase
MCP-1	Monocyte chemoattractant protein 1
mTOR	Mammalian target of rapamycin
NF-κB	Nuclear factor-κB
PI3K	Phosphatidylinositol-4,5-bisphosphate 3-kinase
PPAR	Peroxisome proliferator-activated receptors
PP2A	Protein phosphatase 2 A
pRb	Phosphorylated retinoblastoma protein
ROS	Reactive oxygen species
STAT3	Signal transducer and activator of transcription 3
TC	Thyroid cancer
TNF-α	Tumor necrosis factor-α

## References

[1] (1998). Clinical guidelines on the identification, evaluation and treatment of overweight and obesity in adults: Executive summary. Expert Panel on the Identification, Evaluation and Treatment of Overweight in Adults. Am. J. Clin. Nutr..

[2] Ng M., Fleming T., Robinson M., Thomson B., Graetz N., Margono C., Mullany E.C., Biryukov S., Abbafati C., Abera S.F. (2014). Global, regional and national prevalence of overweight and obesity in children and adults during 1980–2013: A systematic analysis for the Global Burden of Disease Study 2013. Lancet.

[3] NCD Risk Factor Collaboration (NCD-RisC) (2016). Trends in adult body-mass index in 200 countries from 1975 to 2014: A pooled analysis of 1698 population-based measurement studies with 19·2 million participants. Lancet.

[4] Lu Y., Hajifathalian K., Ezzati M., Woodward M., Rimm E.B., Danaei G., Global Burden of Metabolic Risk Factors for Chronic Diseases Collaboration (BMI Mediated Effects) (2014). Metabolic mediators of the effects of body-mass index, overweight and obesity on coronary heart disease and stroke: A pooled analysis of 97 prospective cohorts with 1·8 million participants. Lancet.

[5] Di Angelantonio E., Bhupathiraju S., Wormser D., Gao P., Kaptoge S., Berrington de Gonzalez A., Cairns B., Huxley R., Jackson C., Global BMI Mortality Collaboration (2016). Body-mass index and all-cause mortality: Individual-participant-data meta-analysis of 239 prospective studies in four continents. Lancet.

[6] Renehan A.G., Tyson M., Egger M., Heller R.F., Zwahlen M. (2008). Body-mass index and incidence of cancer: A systematic review and meta-analysis of prospective observational studies. Lancet.

[7] Bifulco M., Ciaglia E. (2017). Updates on “adiponcosis”: More new incoming evidence strengthening the obesity-cancer link. Eur. J. Intern. Med..

[8] Stone T.W., McPherson M., Gail Darlington L. (2018). Obesity and Cancer: Existing and New Hypotheses for a Causal Connection. EBioMedicine.

[9] Galic S., Oakhill J.S., Steinberg G.R. (2010). Adipose tissue as an endocrine organ. Mol. Cell. Endocrinol..

[10] Arita Y., Kihara S., Ouchi N., Takahashi M., Maeda K., Miyagawa J., Hotta K., Shimomura I., Nakamura T., Miyaoka K. (1999). Paradoxical decrease of an adipose-specific protein, adiponectin, in obesity. Biochem. Biophys. Res. Commun..

[11] Nishida M., Funahashi T., Shimomura I. (2007). Pathophysiological significance of adiponectin. Med. Mol. Morphol..

[12] Dalamaga M., Diakopoulos K.N., Mantzoros C.S. (2012). The role of adiponectin in cancer: A review of current evidence. Endocr. Rev..

[13] Hebbard L., Ranscht B. (2014). Multifaceted roles of adiponectin in cancer. Best Pract. Res. Clin. Endocrinol. Metab..

[14] Katira A., Tan P.H. (2016). Evolving role of adiponectin in cancer-controversies and update. Cancer Biol. Med..

[15] Berg A.H., Combs T.P., Du X., Brownlee M., Scherer P.E. (2001). The adipocyte-secreted protein Acrp30 enhances hepatic insulin action. Nat. Med..

[16] Bermúdez V.J., Rojas E., Toledo A., Rodríguez-Molina D., Vega K., Suárez L., Pacheco M., Canelón R., Arráiz N., Rojas J.V.M. (2013). Single-nucleotide polymorphisms in adiponectin, AdipoR1 and AdipoR2 genes: Insulin resistance and type 2 diabetes mellitus candidate genes. Am. J. Ther..

[17] Simpson F., Whitehead J.P. (2010). Adiponectin-It’s all about the modifications. Int. J. Biochem. Cell Biol..

[18] Wang Y., Lam K.S.L., Chan L., Kok W.C., Lam J.B.B., Lam M.C., Hoo R.C.L., Mak W.W.N., Cooper G.J.S., Xu A. (2006). Post-translational modifications of the four conserved lysine residues within the collagenous domain of adiponectin are required for the formation of its high molecular weight oligomeric complex. J. Biol. Chem..

[19] Waki H., Yamauchi T., Kamon J., Ito Y., Uchida S., Kita S., Hara K., Hada Y., Vasseur F., Froguel P. (2003). Impaired multimerization of human adiponectin mutants associated with diabetes. Molecular structure and multimer formation of adiponectin. J. Biol. Chem..

[20] Chiarugi P., Fiaschi T. (2010). Adiponectin in health and diseases: From metabolic syndrome to tissue regeneration. Expert Opin. Ther. Targets.

[21] Yamauchi T., Kamon J., Waki H., Terauchi Y., Kubota N., Hara K., Mori Y., Ide T., Murakami K., Tsuboyama-Kasaoka N. (2001). The fat-derived hormone adiponectin reverses insulin resistance associated with both lipoatrophy and obesity. Nat. Med..

[22] Nigro E., Scudiero O., Monaco M.L., Palmieri A., Mazzarella G., Costagliola C., Bianco A., Daniele A. (2014). New insight into adiponectin role in obesity and obesity-related diseases. Biomed. Res. Int..

[23] Yamauchi T., Iwabu M., Okada-Iwabu M., Kadowaki T. (2014). Adiponectin receptors: A review of their structure, function and how they work. Best Pract. Res. Clin. Endocrinol. Metab..

[24] Hug C., Wang J., Ahmad N.S., Bogan J.S., Tsao T.-S., Lodish H.F. (2004). T-cadherin is a receptor for hexameric and high-molecular-weight forms of Acrp30/adiponectin. Proc. Natl. Acad. Sci. USA.

[25] Wu L., Van Kaer L. (2013). Contribution of lipid-reactive natural killer T cells to obesity-associated inflammation and insulin resistance. Adipocyte.

[26] Catalán V., Gómez-Ambrosi J., Rodríguez A., Frühbeck G. (2013). Adipose tissue immunity and cancer. Front. Physiol..

[27] Sell H., Eckel J. (2010). Adipose tissue inflammation: Novel insight into the role of macrophages and lymphocytes. Curr. Opin. Clin. Nutr. Metab. Care.

[28] Lengyel E., Makowski L., DiGiovanni J., Kolonin M.G. (2018). Cancer as a Matter of Fat: The Crosstalk between Adipose Tissue and Tumors. Trends Cancer.

[29] Ouchi N., Walsh K. (2007). Adiponectin as an anti-inflammatory factor. Clin. Chim. Acta.

[30] James F.R., Wootton S., Jackson A., Wiseman M., Copson E.R., Cutress R.I. (2015). Obesity in breast cancer--what is the risk factor?. Eur. J. Cancer.

[31] Avgerinos K.I., Spyrou N., Mantzoros C.S., Dalamaga M. (2018). Obesity and cancer risk: Emerging biological mechanisms and perspectives. Metabolism.

[32] Ye J., Jia J., Dong S., Zhang C., Yu S., Li L., Mao C., Wang D., Chen J., Yuan G. (2014). Circulating adiponectin levels and the risk of breast cancer: A meta-analysis. Eur. J. Cancer Prev..

[33] Mauro L., Pellegrino M., De Amicis F., Ricchio E., Giordano F., Rizza P., Catalano S., Bonofiglio D., Sisci D., Panno M.L. (2014). Evidences that estrogen receptor α interferes with adiponectin effects on breast cancer cell growth. Cell Cycle.

[34] Liu J., Lam J.B.B., Chow K.H.M., Xu A., Lam K.S.L., Moon R.T., Wang Y. (2008). Adiponectin stimulates Wnt inhibitory factor-1 expression through epigenetic regulations involving the transcription factor specificity protein 1. Carcinogenesis.

[35] Otvos L., Knappe D., Hoffmann R., Kovalszky I., Olah J., Hewitson T.D., Stawikowska R., Stawikowski M., Cudic P., Lin F. (2014). Development of second generation peptides modulating cellular adiponectin receptor responses. Front. Chem..

[36] Mauro L., Naimo G.D., Ricchio E., Panno M.L., Andò S. (2015). Cross-Talk between Adiponectin and IGF-IR in Breast Cancer. Front. Oncol..

[37] Di Zazzo E., Feola A., Zuchegna C., Romano A., Donini C.F., Bartollino S., Costagliola C., Frunzio R., Laccetti P., Di Domenico M. (2014). The p85 regulatory subunit of PI3K mediates cAMP-PKA and insulin biological effects on MCF-7 cell growth and motility. Sci. World J..

[38] Donini C.F., Coppa A., Di Zazzo E., Zuchegna C., Di Domenico M., D’Inzeo S., Nicolussi A., Avvedimento E.V., Coppa A., Porcellini A. (2012). The p85α regulatory subunit of PI3K mediates cAMP-PKA and retinoic acid biological effects on MCF7 cell growth and migration. Int. J. Oncol..

[39] Di Zazzo E., Bartollino S., Moncharmont B. (2017). The master regulator gene PRDM2 controls C2C12 myoblasts proliferation and Differentiation switch and PRDM4 and PRDM10 expression. Insights Biol. Med..

[40] Mauro L., Pellegrino M., Giordano F., Ricchio E., Rizza P., De Amicis F., Catalano S., Bonofiglio D., Panno M.L., Andò S. (2015). Estrogen receptor- *α* drives adiponectin effects on cyclin D1 expression in breast cancer cells. FASEB J..

[41] Jia Z., Liu Y., Cui S. (2014). Adiponectin Induces Breast Cancer Cell Migration and Growth Factor Expression. Cell Biochem. Biophys..

[42] Jeong Y.J., Bong J.G., Park S.H., Choi J.H., Oh H.K. (2011). Expression of leptin, leptin receptor, adiponectin and adiponectin receptor in ductal carcinoma in situ and invasive breast cancer. J. Breast Cancer.

[43] Pfeiler G., Treeck O., Wenzel G., Goerse R., Hartmann A., Schmitz G., Ortmann O. (2009). Influence of insulin resistance on adiponectin receptor expression in breast cancer. Maturitas.

[44] De Groot P., Munden R.F. (2012). Lung cancer epidemiology, risk factors and prevention. Radiol. Clin. N. Am..

[45] Hidayat K., Du X., Chen G., Shi M., Shi B. (2016). Abdominal Obesity and Lung Cancer Risk: Systematic Review and Meta-Analysis of Prospective Studies. Nutrients.

[46] Yang Y., Dong J., Sun K., Zhao L., Zhao F., Wang L., Jiao Y. (2013). Obesity and incidence of lung cancer: A meta-analysis. Int. J. Cancer.

[47] Daniele A., De Rosa A., Nigro E., Scudiero O., Capasso M., Masullo M., de Laurentiis G., Oriani G., Sofia M., Bianco A. (2012). Adiponectin oligomerization state and adiponectin receptors airway expression in chronic obstructive pulmonary disease. Int. J. Biochem. Cell Biol..

[48] Petridou E.T., Mitsiades N., Gialamas S., Angelopoulos M., Skalkidou A., Dessypris N., Hsi A., Lazaris N., Polyzos A., Syrigos C. (2007). Circulating adiponectin levels and expression of adiponectin receptors in relation to lung cancer: Two case-control studies. Oncology.

[49] Gulen S.T., Karadag F., Karul A.B., Kilicarslan N., Ceylan E., Kuman N.K., Cildag O. (2012). Adipokines and Systemic Inflammation in Weight-Losing Lung Cancer Patients. Lung.

[50] Karapanagiotou E.M., Tsochatzis E.A., Dilana K.D., Tourkantonis I., Gratsias I., Syrigos K.N. (2008). The significance of leptin, adiponectin and resistin serum levels in non-small cell lung cancer (NSCLC). Lung Cancer.

[51] Denzel M.S., Scimia M.-C., Zumstein P.M., Walsh K., Ruiz-Lozano P., Ranscht B. (2010). T-cadherin is critical for adiponectin-mediated cardioprotection in mice. J. Clin. Investig..

[52] Cui E., Deng A., Wang X., Wang B., Mao W., Feng X., Hua F. (2011). The role of adiponectin (ADIPOQ) gene polymorphisms in the susceptibility and prognosis of non-small cell lung cancer. Biochem. Cell Biol..

[53] Nigro E., Scudiero O., Sarnataro D., Mazzarella G., Sofia M., Bianco A., Daniele A. (2013). Adiponectin affects lung epithelial A549 cell viability counteracting TNFα and IL-1ß toxicity through AdipoR1. Int. J. Biochem. Cell Biol..

[54] Illiano M., Nigro E., Sapio L., Caiafa I., Spina A., Scudiero O., Bianco A., Esposito S., Mazzeo F., Pedone P.V. (2017). Adiponectin down-regulates CREB and inhibits proliferation of A549 lung cancer cells. Pulm. Pharmacol. Ther..

[55] Chen H.-M., Yang C.-M., Chang J.-F., Wu C.-S., Sia K.-C., Lin W.-N. (2016). AdipoR-increased intracellular ROS promotes cPLA2 and COX-2 expressions via activation of PKC and p300 in adiponectin-stimulated human alveolar type II cells. Am. J. Physiol. Lung Cell. Mol. Physiol..

[56] Tarasiuk A., Mosińska P., Fichna J. (2018). The mechanisms linking obesity to colon cancer: An overview. Obes. Res. Clin. Pract..

[57] Riondino S., Roselli M., Palmirotta R., Della-Morte D., Ferroni P., Guadagni F. (2014). Obesity and colorectal cancer: Role of adipokines in tumor initiation and progression. World J. Gastroenterol..

[58] Moon H.-S., Liu X., Nagel J.M., Chamberland J.P., Diakopoulos K.N., Brinkoetter M.T., Hatziapostolou M., Wu Y., Robson S.C., Iliopoulos D. (2013). Salutary effects of adiponectin on colon cancer: In vivo and in vitro studies in mice. Gut.

[59] Saetang J., Boonpipattanapong T., Palanusont A., Maneechay W., Sangkhathat S. (2016). Alteration of Leptin and Adiponectin in Multistep Colorectal Tumorigenesis. Asian Pac. J. Cancer Prev..

[60] Xu X.T., Xu Q., Tong J.L., Zhu M.M., Huang M.L., Ran Z.H., Xiao S.D. (2011). Meta-analysis: Circulating adiponectin levels and risk of colorectal cancer and adenoma. J. Dig. Dis..

[61] Tae C.H., Kim S.-E., Jung S.-A., Joo Y.-H., Shim K.-N., Jung H.-K., Kim T.H., Cho M.-S., Kim K.H., Kim J.S. (2014). Involvement of adiponectin in early stage of colorectal carcinogenesis. BMC Cancer.

[62] Kumor A., Daniel P., Pietruczuk M., Małecka-Panas E. (2009). Serum leptin, adiponectin and resistin concentration in colorectal adenoma and carcinoma (CC) patients. Int. J. Colorectal Dis..

[63] Ayyildiz T., Dolar E., Ugras N., Adim S.B., Yerci O. (2014). Association of adiponectin receptor (Adipo-R1/-R2) expression and colorectal cancer. Asian Pac. J. Cancer Prev..

[64] Ren J.-Z., Huo J.-R. (2012). Correlation between T-cadherin gene expression and aberrant methylation of T-cadherin promoter in human colon carcinoma cells. Med. Oncol..

[65] Ye M., Huang T., Li J., Zhou C., Yang P., Ni C., Chen S. (2017). Role of CDH13 promoter methylation in the carcinogenesis, progression and prognosis of colorectal cancer: A systematic meta-analysis under PRISMA guidelines. Medicine.

[66] Choe E.K., Yi J.W., Chai Y.J., Park K.J. (2018). Upregulation of the adipokine genes ADIPOR1 and SPP1 is related to poor survival outcomes in colorectal cancer. J. Surg. Oncol..

[67] Sugiyama M., Takahashi H., Hosono K., Endo H., Kato S., Yoneda K., Nozaki Y., Fujita K., Yoneda M., Wada K. (2009). Adiponectin inhibits colorectal cancer cell growth through the AMPK/mTOR pathway. Int. J. Oncol..

[68] Kim A.Y., Lee Y.S., Kim K.H., Lee J.H., Lee H.K., Jang S.-H., Kim S.-E., Lee G.Y., Lee J.-W., Jung S.-A. (2010). Adiponectin represses colon cancer cell proliferation via AdipoR1- and -R2-mediated AMPK activation. Mol. Endocrinol..

[69] Davis J.E., Gabler N.K., Walker-Daniels J., Spurlock M.E. (2008). Tlr-4 deficiency selectively protects against obesity induced by diets high in saturated fat. Obesity.

[70] Priego T., Sánchez J., Picó C., Palou A. (2008). Sex-differential expression of metabolism-related genes in response to a high-fat diet. Obesity.

[71] Boddicker R.L., Whitley E.M., Davis J.E., Birt D.F., Spurlock M.E. (2011). Low-dose dietary resveratrol has differential effects on colorectal tumorigenesis in adiponectin knockout and wild-type mice. Nutr. Cancer.

[72] Hwang J.-T., Ha J., Park I.-J., Lee S.-K., Baik H.W., Kim Y.M., Park O.J. (2007). Apoptotic effect of EGCG in HT-29 colon cancer cells via AMPK signal pathway. Cancer Lett..

[73] Nigro E., Schettino P., Polito R., Scudiero O., Monaco M.L., De Palma G.D., Daniele A. (2018). Adiponectin and colon cancer: Evidence for inhibitory effects on viability and migration of human colorectal cell lines. Mol. Cell. Biochem..

[74] Saxena A., Chumanevich A., Fletcher E., Larsen B., Lattwein K., Kaur K., Fayad R. (2012). Adiponectin deficiency: Role in chronic inflammation induced colon cancer. Biochim. Biophys. Acta Mol. Basis Dis..

[75] Ogunwobi O.O., Beales I.L.P. (2006). Adiponectin stimulates proliferation and cytokine secretion in colonic epithelial cells. Regul. Pept..

[76] Habeeb B.S., Kitayama J., Nagawa H. (2011). Adiponectin supports cell survival in glucose deprivation through enhancement of autophagic response in colorectal cancer cells. Cancer Sci..

[77] Dossus L., Franceschi S., Biessy C., Navionis A.-S., Travis R.C., Weiderpass E., Scalbert A., Romieu I., Tjønneland A., Olsen A. (2018). Adipokines and inflammation markers and risk of differentiated thyroid carcinoma: The EPIC study. Int. J. Cancer.

[78] Abooshahab R., Yaghmaei P., Ghadaksaz H.G., Hedayati M. (2016). Lack of Association between Serum Adiponectin/Leptin Levels and Medullary Thyroid Cancer. Asian Pac. J. Cancer Prev..

[79] Cheng S.-P., Liu C.-L., Hsu Y.-C., Chang Y.-C., Huang S.-Y., Lee J.-J. (2013). Expression and biologic significance of adiponectin receptors in papillary thyroid carcinoma. Cell Biochem. Biophys..

[80] Lin S.-Y., Huang S.-C., Sheu W.H.-H. (2010). Circulating adiponectin concentrations were related to free thyroxine levels in thyroid cancer patients after thyroid hormone withdrawal. Metabolism.

[81] Mitsiades N., Pazaitou-Panayiotou K., Aronis K.N., Moon H.-S., Chamberland J.P., Liu X., Diakopoulos K.N., Kyttaris V., Panagiotou V., Mylvaganam G. (2011). Circulating adiponectin is inversely associated with risk of thyroid cancer: In vivo and in vitro studies. J. Clin. Endocrinol. Metab..

[82] Kim W.G., Park J.W., Willingham M.C., Cheng S. (2013). Diet-induced obesity increases tumor growth and promotes anaplastic change in thyroid cancer in a mouse model. Endocrinology.

[83] Liao W., Yu C., Wen J., Jia W., Li G., Ke Y., Zhao S., Campell W. (2009). Adiponectin induces interleukin-6 production and activates STAT3 in adult mouse cardiac fibroblasts. Biol. Cell.

[84] Disney-Hogg L., Sud A., Law P.J., Cornish A.J., Kinnersley B., Ostrom Q.T., Labreche K., Eckel-Passow J.E., Armstrong G.N., Claus E.B. (2018). Influence of obesity-related risk factors in the aetiology of glioma. Br. J. Cancer.

[85] Porcile C., Di Zazzo E., Monaco M.L., D’Angelo G., Passarella D., Russo C., Di Costanzo A., Pattarozzi A., Gatti M., Bajetto A. (2014). Adiponectin as novel regulator of cell proliferation in human glioblastoma. J. Cell. Physiol..

[86] Di Zazzo E., Galasso G., Giovannelli P., Di Donato M., Castoria G. (2018). Estrogens and Their Receptors in Prostate Cancer: Therapeutic Implications. Front. Oncol..

